# Peripheral T-cell Lymphoma, Not Otherwise Specified: An Unusual Presentation of a Rare Lymphoma

**DOI:** 10.7759/cureus.3813

**Published:** 2019-01-02

**Authors:** Aditi Singh, Milind Bhagat, Ahmad D Siddiqui, Simant S Thapa

**Affiliations:** 1 Internal Medicine, St. Vincent Hospital, Worcester, USA; 2 Internal Medicine, Rhode Island Hospital, Providence, USA; 3 Hematology and Oncology, St. Vincent Hospital, Worcester, USA

**Keywords:** lymphomas, rash, peripheral t-cell lymphoma not otherwise specified (ptcl-nos)

## Abstract

We present a case of peripheral T-cell lymphoma, not otherwise specified (PTCL-NOS) masquerading as a skin rash which progressively worsened over a year. After being treated for various dermatologic and infectious etiologies, he did not feel any relief and presented to our hospital. Imaging showed generalized lymphadenopathy. Later, lymph node biopsy and skin biopsy confirmed the diagnosis of CD30 + peripheral T-cell lymphoma. He was soon started on chemotherapy with cyclophosphamide, doxorubicin, etoposide, vincristine, and prednisone (CHOEP). However, because of the aggressive nature of his disease and advanced stage at presentation, he succumbed to complications and died of sepsis.

This case highlights the importance of considering a rash as one of the early symptoms of an underlying life-threatening disease.

## Introduction

Peripheral T-cell lymphoma, not otherwise specified (PTCL-NOS) alludes to a heterogeneous group of mature T-cell lymphomas, features of which do not correspond to any of the other well-defined categories [1]. PTCL-NOS accounts for about one in four cases of PTCL. It is the most common subtype of PTCL in North America and Europe, as compared to natural killer (NK) T-cell lymphoma and adult T-cell leukemia/lymphoma, which are more common in Asia. It is more common in males, with a mean age of presentation around 60 years of age, and the majority presenting with an advanced stage [2]. PTCL directly involves the skin in only a minority of the cases [3-4].

PTCL-NOS has a five-year survival of approximately 32% and five-year failure-free survival of 20% [5]. Considering that a majority of patients are at an advanced stage at the time of diagnosis and considering the overall aggressiveness of the tumor, a favorable prognosis is highly dependent on early diagnosis. 

Our patient presented with a very common dermatologic symptom (rash) which, although hiding in plain sight, can potentially result in the loss of valuable treatment time in arriving at the correct diagnosis. 

## Case presentation

A 54-year-old Caucasian man presented to the hospital with a rash of one year's duration. He had no significant past medical history, apart from moderate daily beer intake and one-pack-per-day cigarette smoking. A pruritic maculopapular rash first developed in his left lower extremity and later became generalized. He had been so far treated for scabies, dry skin, allergies, and cellulitis. He had visited multiple urgent care clinics, dermatologists, and infectious disease specialists without any solution to his predicament. Skin biopsies had only shown external trauma and excoriations. His ambiguous disease had now caused him dysphagia and weight loss. 

**Figure 1:**
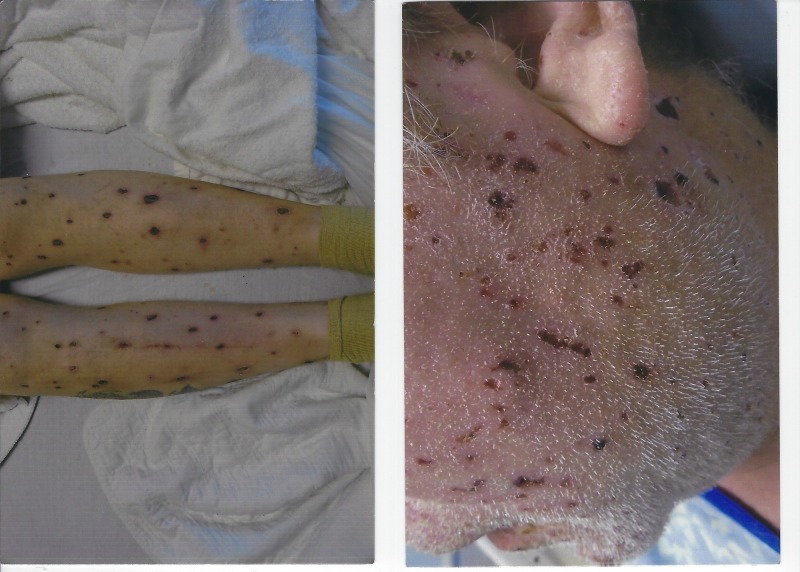
PTCL-NOS involvement in skin causing skin lesions seen on the legs and face PTCL-NOS: peripheral T-cell lymphoma, not otherwise specified

His initial vital signs revealed a blood pressure of 115/78 mmHg, heart rate 120/m, sinus rhythm, respiratory rate of 18/m, temperature 98.1° F, and oxygenation saturation of 98% on room air. On examination, he was found to have a purulent nasal discharge, oropharyngeal thrush, tonsillar enlargement, foul breath, and maxillary sinus tenderness. He had generalized lymphadenopathy. Skin examination revealed a generalized rash involving the oral mucosa, face, palms, and soles. The rash was papulosquamous on his back. He had coin-shaped lesions on his legs and arms (Figure 1). The skin on the palm and soles was macerated. 

Investigation

Laboratory studies revealed a normal complete blood count, basic metabolic panel, and liver function tests. Blood cultures were negative. The human immunodeficiency virus (HIV), rapid plasma reagin (RPR), herpes zoster culture, a fungal antigen, rheumatoid factor (RF), antinuclear antibody (ANA), cytoplasmic antineutrophil cytoplasmic antibody (C-ANCA), perinuclear antineutrophil cytoplasmic antibody (P-ANCA), and atypical P-ANCA were all negative. 

Computed tomography scans of the chest, abdomen, and pelvis with contrast, obtained to further evaluate the generalized lymphadenopathy, showed the extensive burden of adenopathy in bilateral axillary regions, mediastinum, retroperitoneum, and both iliac chains into the inguinal regions. 

Duodenal compression at the third and fourth segment was seen with possible additional wall-thickening, raising concern for lymphomatous involvement.

Diagnosis

**Figure 2:**
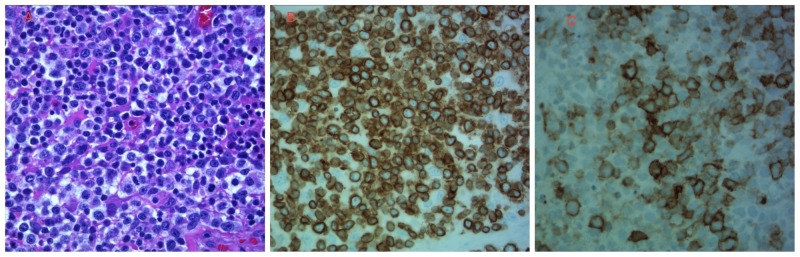
Histopathology from the lymph node biopsy A) hematoxylin-eosin (H&E) peripheral T-cell lymphoma, not otherwise specified (PTCL-NOS) composed of pleomorphic medium to large cells with clear cytoplasm and nuclear irregularities admixed with reactive cells; B) tumor cells positive for CD3 immunostain with membranous staining; C) tumor cells positive for CD30 immunostain with membranous and Golgi-type staining.

Inguinal lymph node and skin biopsies were done. The inguinal lymph node biopsy revealed extensive tumor necrosis. Immunohistochemical stains were positive for CD3, CD30, and BCL2 and were negative for EMA, CD 20, CD45, and PAX5 (Figure 2). Flow cytometry showed a large population of abnormal T-cells which made up 88% of the total cells. These cells were moderately positive for CD2, CD3, and CD5, weakly positive for T-cell receptor (TCR) alpha/beta, and partially positive for CD11c, CD25, CD30, and CD57. They aberrantly lacked CD7 and were CD4 and CD8 dual-negative. CD56 was also negative. The CD3 positive normal T-cells was 9% of the total cells without the loss of pan-T-cell antigens. The CD4: CD8 ratio was 1.6:1. The CD19 and CD20 positive B-cells were 1% of total cells. It was consistent with CD30-positive peripheral T-cell lymphoma.

A skin biopsy from the right thigh showed atypical T-cell lymphoid infiltrate, including many CD30+ cells. Under the acanthotic and partially ulcerated epidermis, there was a dense monotonous lymphocyte infiltrate in the dermis. The lymphocytes showed significant cytologic atypia and perivascular accentuation. Immunostains showed the infiltrates were composed of mostly CD3+ T-lymphocytes with an increased CD4/CD8 ratio and reduced CD7 expression. Many of these lymphocytes were CD30+. There were also numerous CD1a+ dendritic cells in the epidermis and CD68+ histiocytes in the dermis. Only a few CD20+ B-lymphocytes and CD138+ plasma cells were present. Eosinophils were absent, and no evidence of vasculitis was seen.   

The above histopathological findings and the immunophenotypical results, along with the patient's history of lymph node CD30+ lymphoma was consistent with the cutaneous involvement of CD30+ T-cell lymphoma.   

A bone marrow biopsy did not show the involvement of lymphoma. He was diagnosed with Stage IV PTCL-NOS. 

Management

The patient was started on chemotherapy with cyclophosphamide, doxorubicin, etoposide, vincristine, and prednisone (CHOEP). He had improvement in the skin rash and lymphadenopathy after two cycles. His disease course was complicated by gastrointestinal bleeding from a duodenal perforation. He did not survive to finish the planned course of chemotherapy and succumbed to sepsis and respiratory failure. 

## Discussion

PTCL-NOS as a category of lymphomas was first mentioned in 1994 in the Revised European-American Lymphoma (REAL) classification as PTCL-unspecified, which included various groups of mature T-cell lymphomas that did not fit into any of the established groups [6]. This has been updated to the currently known PTCL-NOS group in the World Health Organization (WHO) classification [6-7]. As a growing number of non-mycosis fungoides, cutaneous T-cell lymphomas have been recognized in recent years, the frequency of peripheral T-cell lymphoma (primary cutaneous or combined systemic and cutaneous) has been decreasing. We present a rare case with combined cutaneous and systemic features at diagnosis of PTCL-NOS with CD4- CD8- (double negative) (rarer) to examine the clinical and histopathological features of this disease.

In the prognostic analysis done by Tolkachjov et al., 15 out of 29 patients were found to have concurrent cutaneous and systemic involvement among those diagnosed to have PTCL-NOS. Clinical lesions of the dermatological presentation included plaques (10 patients), papules (three patients), nodules (three patients), tumors (one patient), and patches (two patients), with four patients having > 1 morphology. Ulceration of the lesions, B-symptoms at presentation, and multifocal lesions suggest concurrent systemic and cutaneous PTCL-NOS [8].

The proposed normal counterpart cells seen in PTCL-NOS are the CD4 central memory type T-lymphocytes. The cytology seen is often pleomorphic and seen as a mix of small and large cells with a high proliferation rate. It has been divided into three morphologic variants as per the WHO 2008 classification, namely, lymphoepitheloid (known as Lennert’s type), follicular, and T-zone. Lennert’s variant is mainly characterized by the predominance of reactive epithelial histiocytes. The follicular variant is so called because of its similarity to follicular lymphoma, has an occasional association with a t(5;9) (q33;q22) translocation, and is now classified as one of the nodal T-cell lymphomas with a T-follicular helper phenotype. The T-zone variant is so called due to its growth pattern in the T-zone of lymph nodes [7]. 

Phenotypically, PTCL-NOS shows expressivity for most of the T-cell antigens which include CD3, CD5, CD2, and CD7 with loss of any one antigen in four out of five cases. It originates usually from CD4 cells, but rare cases can be just CD8+, both CD4 and CD8+, or CD4- CD8- (as seen in this patient) [9].

An analysis done by Bekkenk et al. on 82 patients showed that peripheral T-cell lymphomas, unspecified, presenting in the skin have an unfavorable prognosis [8]. The prognostic marker used for PTCL-NOS is called the prognostic index for T-cell lymphoma, and it consists of age, elevated lactate dehydrogenase (LDH), poor performance status at baseline, and bone marrow involvement. The median survival for concurrent PTCL-NOS is around 2.1 years, and the time from symptom onset to diagnosis has been inversely associated with survival [10].

There is no consensus about optimal therapy for T- and NK-cell neoplasms. Cyclophosphamide, doxorubicin, vincristine, and prednisone (CHOP) is considered the standard therapy in PTCL-NOS [11]. The addition of etoposide, like in the index case, has not shown any significant improvement in overall survival cure rates. In some studies, the addition of etoposide to CHOP showed an improvement in event-free survival and progression-free survival [12-13].

## Conclusions

We attempt to raise awareness about considering rash as a differential diagnosis for underlying malignancy, especially when the rash is of prolonged duration and the overall condition of patient deteriorates. A timely diagnosis of underlying aggressive malignancy, as in this case, can affect the subsequent outcome. 

## References

[1] Al-Zahrani M, Savage KJ (2017). Peripheral T-cell lymphoma, not otherwise specified: A review of current disease understanding and therapeutic approaches. Hematol Oncol Clin North Am.

[2] Abouyabis AN, Shenoy PJ, Lechowicz MJ, Flowers CR (2008). Incidence and outcomes of the peripheral T-cell lymphoma subtypes in the United States. Leuk Lymphoma.

[3] Rodriguez-Abreu D, Filho VB, Zucca E (2008). Peripheral T-cell lymphomas, unspecified (or not otherwise specified): a review. Hematol Oncol.

[4] Schowalter MK, Akilov OE, Story SK, Geskin LJ (2012). Cutaneous manifestations of unspecified peripheral T-cell lymphoma may be indicative of disease activity and predict response to therapy. J Clin Oncol.

[5] Weisenburger DD, Savage KJ, Harris NL (2011). Peripheral T-cell lymphoma, not otherwise specified: a report of 340 cases from the International Peripheral T-cell Lymphoma Project. Blood.

[6] Kim K, Kim WS, Jung CW (2002). Clinical features of peripheral T-cell lymphomas in 78 patients diagnosed according to the Revised European-American lymphoma (REAL) classification. Eur J Cancer.

[7] Swerdlow SH, Campo E, Pileri SA (2016). The 2016 revision of the World Health Organization classification of lymphoid neoplasms. Blood.

[8] Tolkachjov SN, Weenig RH, Comfere NI (2016). Cutaneous peripheral T-cell lymphoma, not otherwise specified: a single-center prognostic analysis. J Am Acad Dermatol.

[9] Vose J, Armitage J, Weisenburger D; International T-Cell Lymphoma Project (2008). International peripheral T-cell and natural killer/T-cell lymphoma study: pathology findings and clinical outcomes. J Clin Oncol.

[10] Bekkenk MW1, Vermeer MH, Jansen PM (2003). Peripheral T-cell lymphomas unspecified presenting in the skin: analysis of prognostic factors in a group of 82 patients. Blood.

[11] Dearden CE, Johnson R, Pettengell R (2011). Guidelines for the management of mature T-cell and NK-cell neoplasms (excluding cutaneous T-cell lymphoma). Br J Haematol.

[12] Ellin F, Landström J, Jerkeman M, Relander T (2014). Real-world data on prognostic factors and treatment in peripheral T-cell lymphomas: a study from the Swedish Lymphoma Registry. Blood.

[13] Schmitz N, Trümper L, Ziepert M (2010). Treatment and prognosis of mature T-cell and NK-cell lymphoma: an analysis of patients with T-cell lymphoma treated in studies of the German High-Grade Non-Hodgkin Lymphoma Study Group. Blood.

